# Consumption of *in natura* and ultra-processed foods in adults: an analysis of social, metabolic, and lifestyle determinants

**DOI:** 10.1590/1980-549720240018

**Published:** 2024-04-29

**Authors:** Renata Kelly Gomes Oliveira, Ivanildo Ribeiro Domingos, Vanessa Sá Leal, Juliana Souza Oliveira, Pedro Israel Cabral de Lira, Nathália Paula de Souza

**Affiliations:** I Universidade Federal de Pernambuco, Centro Acadêmico de Vitória, Nutrition Center – Vitória de Santo Antão (PE), Brazil.; II Universidade Federal de Pernambuco, Health Sciences Center, Department of Nutrition – Recife (PE), Brazil.

**Keywords:** Food consumption, Ultra-processed foods, In natura foods, Social determinants of health, Consumo alimentar, Alimentos ultraprocessados, Alimentos *in natura*, Determinantes sociais da saúde

## Abstract

**Objective:**

To assess the social, metabolic, and lifestyle determinants of consumption of fruits, vegetables, and greens (FVG) and ultra-processed food (ULT) in adults from Pernambuco.

**Methods:**

Cross-sectional and analytical study, conducted in 2015/2016. In addition to sociodemographic variables, the determinants of lifestyle were level of physical activity, alcohol consumption, tobacco use, and metabolic variables were self-reported hypertension, blood glucose, and Body Mass Index (BMI). Consumption was measured by the Food Frequency Questionnaire, then created the Frequency of Consumption Index (SFI) of the mean intake of ULT and FVG foods. The indices of FVG and ULT consumption were transformed into quartiles and these variables were included in the multinomial logistic regression, considering their determinants when p<0.05.

**Results:**

The sample was representative of the state, with 1,067 people being interviewed, whose intake of ULT was higher than that of FVG in the lowest and highest quartile of the consumption index. Consumption of fruit and vegetables was higher in higher consumption of alcoholic beverages (p=0.031) and BMI>25 kg/m^2^ (p=0.047); and lower in the lowest income (p=0.001). ULT intake was higher in young adults (p=0.005), lower income (p=0.044), and controlled blood glucose (p=0.021). Rural areas were 52% less exposed to medium-high ULT consumption (p<0.006).

**Conclusion:**

Higher rate of ULT consumption in relation to fresh foods, with income as a common determinant, inversely associated with ULT intake and directly related to FVG, which demands structuring policies.

## RESUMO

**Objetivo:**

Avaliar os determinantes sociais, metabólicos e de estilo de vida do consumo de frutas, legumes e verduras (FLV) e ultraprocessados (ULT) em adultos de Pernambuco.

**Métodos:**

Estudo transversal e analítico, realizado em 2015/2016. Além de variáveis sociodemográficas, os determinantes do estilo de vida foram nível de atividade física, doses de bebida alcoólica e uso de tabaco e os metabólicos foram hipertensão autorreferida, glicemia e Índice de Massa Corporal (IMC). O consumo foi mensurado por questionário de frequência alimentar, e, em seguida, criou-se Índice da Frequência de Consumo (IFC) da média de ingestão dos alimentos ULT e FVL. Os índices de consumo de FLV e ULT foram transformados em quartis e essas variáveis incluídas na regressão logística multinomial, considerando seus determinantes quando p<0,05.

**Resultados:**

A amostra foi representativa do estado, sendo entrevistadas 1.067 pessoas, cuja ingestão de ULT foi superior à de FVL no menor e no maior quartil do índice de consumo. O consumo de FLV foi superior no maior consumo de bebida alcoólica (p=0,031) e IMC>25 kg/m^2^ (p=0,047); e inferior na menor renda (p=0,001). O consumo de ULT foi maior em adultos jovens (p=0,005), menor renda (p=0,044) e glicemia controlada (p=0,021). A área rural estava 52% menos exposta ao consumo médio-alto de ULT (p<0,006).

**Conclusão:**

Maior índice de consumo de ULT em relação aos alimentos *in natura*, tendo a renda como um determinante comum, inversamente associada à ingestão de ULT e diretamente relacionada a FVL, o que demanda políticas estruturantes.

## INTRODUCTION

Noncommunicable chronic diseases (NCDs) represent one of the most significant global public health challenges today. According to estimates from the World Health Organization (WHO), NCDs account for 71% of the 57 million deaths worldwide^1^. In Brazil, these figures reached 74% of total deaths in 2016, with emphasis on cardiovascular diseases with around 28%^1^.

Data from the Surveillance of Risk and Protective Factors for Chronic Diseases by Telephone Survey (*Vigilância de Fatores de Risco e Proteção para Doenças Crônicas por Inquérito Telefônico* – Vigitel) show that the prevalence of diabetes increased from 5.5 to 9.1%, between 2006 and 2021; arterial hypertension, from 22.6 to 26.3%; and obesity showed an increase of approximately 90%, from 11.8 to 22.4%, in both genders^2,3^.

GBD 2017 Diet Collaborators, in their analysis of food and nutrient consumption across 195 countries, revealed that, in 2017, dietary risk factors, including high sodium intake and low consumption of whole grains and fruits, contributed to 11 million deaths and 255 million disability-adjusted life years^4^. High sodium intake often correlates with the increase in consumption of ultra-processed foods over natural and minimally processed alternatives^5^, which changes traditional dietary patterns and can lead to health problems^6^.

Ultra-processed foods undergo numerous processing steps and techniques, often involving the addition of salt, sugar, oils, fats, as well as a variety of additives that alter sensory attributes such as emulsifiers, dyes, flavorings, etc. That is to say that these products are primarily composed of substances derived from foods and additives, with minimal or no intact food content^7^. Studies indicate that the intake of ultra-processed foods is associated with a greater risk of obesity^8^, hypertension^9^, and type 2 *diabetes mellitus* ^10^. Conversely, diets rich in fruits and vegetables contribute to maintaining overall health and body weight^11^, reducing the risk of developing NCDs.

Education, income, type of employment^12^, calorie intake, lifestyle habits^13^, and demographic and economic disparities^14^ are intricately connected to dietary quality, although there is no unanimity about this relationship in the literature. Therefore, it is imperative to explore living standards and dietary habits to gain deeper understanding of their implications for health and disease. This knowledge may provide valuable insights on subsidies, planning, and organization of services, as well as formulate policies and actions within the scope of public health^15^.

Due to this bias, the present study aimed to evaluate the social, metabolic, and lifestyle determinants of the consumption of fruits/vegetables/greens and ultra-processed foods in adults in the state of Pernambuco, Brazil, in the 2015/2016 biennium in order to better understand how dietary patterns and socioeconomic issues are associated with the occurrence of NCDs.

## METHODS

This study is a cross-sectional and analytical investigation based on the IV State Health and Nutrition Survey (*Pesquisa Estadual de Saúde e Nutrição* – PESN), carried out during the 2015/2016 biennium, in Pernambuco, which is a population survey employing a random sample of households from urban and rural areas, coordinated by the Laboratory of Nutrition in Public Health of the Department of Nutrition of Universidade Federal de Pernambuco (UFPE), whose primary objectives include estimating the prevalence and risk factors for NCDs in the adult population and evaluating structural and functional conditions of public health services.

The study population consisted of adults of both genders, aged between 20 and 59 years. The sample was representative of the state of Pernambuco, with sample calculations conducted using the Statcalc program of the EPI-Info software, version 6.04 (Centers for Disease Control and Prevention, Atlanta, United States). The mean prevalence of daily consumption of fruits (17.4%), vegetables (9.2%), and greens (20.3%) was used, equal to 15.6%, in Pernambuco, obtained in the III PESN16. A confidence interval of 95% and a sampling error of ±2.3% were adopted, estimating the sample at 955 individuals. An additional 10% was added to account for potential losses, bringing the total anticipated participants to approximately 1,062.

The sampling plan was stratified into four stages:

1. Municipalities;
2. Census tracts (CT);
3. Households; and
4. Individuals.

The selection of municipalities was based on data from the III PESN (2006) (n=13), and CTs were selected randomly and without replacement, using records from the 2010 Demographic Census. A total of 29 CTs were selected, from which 40 households were drawn. Within each household, one adult was randomly invited to participate in the research.

The sociodemographic variables included in this study were: age (grouped into ranges of 20–29.99; 30–39; 40–49; 50–59); gender (female and male); housing area (rural; urban); number of people in the household (up to four people; five or more); water treatment (filtered/boiled/strained; untreated; mineral); waste destination (general network; covered pit; others); level of education by years of study (greater than or equal to complete high school; complete elementary school/incomplete high school; never attended to school/incomplete elementary school); *per capita* family income, in minimum wage (MW) (less than 0.5 MW; 0.5–1.0 MW; ≥1 MW). To calculate *per capita* income, the number of people living in the household was taken into account, and the minimum wage corresponds to the year in which data collection was carried out, that is, in 2015, R$ 788.00, and in 2016, R$ 865.50.

Regarding lifestyle, the level of physical activity, alcohol consumption, smoking and dietary patterns were assessed. Parameters of physical activity practice were identified using the short version of the International Physical Activity Questionnaire (Ipaq)^17^ and classified according to criteria established by the WHO (2010): very active, when engaged in vigorous intensity activity for, at least, 150 minutes or 60 minutes plus moderate walking for, at least, 150 minutes per week; active, when engaging in, at least, 60 minutes of vigorous intensity or 150 minutes of moderate intensity activity or walking or equivalent combination of both throughout the week or any activity combined with, at least, 150 minutes per week; irregularly active, when physical activity was insufficient to be classified as active and sedentary when they did not perform any physical activity for, at least, 10 continuous minutes during the week^18^.

Regarding alcoholic beverages, the Brazilian Society of Cardiology (*Sociedade Brasileira de Cardiologia* – SBC) cutoff point was adopted, which considers daily consumption to be high when it exceeds one dose for women and two doses for men, corresponding to approximately 15 and 30 g of ethanol, respectively. A daily dose corresponds to around 350 ml of beer (a can), 150 ml of wine (a glass), and 45 ml of distilled beverage (a “dose”)^19^. As for smoking, individuals who reported using any type of cigarette, whether at the time of the survey or in a previous period, were classified as smokers^19^.

Food consumption was assessed using a food frequency questionnaire (FFQ), developed by Furlan-Viebig and Pastor-Valero^20^. This questionnaire is considered semi-quantitative and comprises 123 food items. It provides standardized consumption portions, based on the average portions reported by participants in the recalls, such as “five units of salted biscuit or two pieces of pizza.” It includes nine possible response categories, ranging from “never” to “more than six times/day.”

For better categorization, only foods consumed equal to or greater than once a week or four to seven times a month were selected, totaling 10 items for the fruit, vegetables, and greens (FVG) group and eight items for the ultra-processed (ULT) group. The FVG group included banana, orange, passion fruit, acerola, apple, watermelon, papaya, raw salad, carrot, and pumpkin. ULT consisted of margarine, cream cracker biscuits, cornstarch or maria biscuits, sausages, candies and sweets, biscuits with filling or butter, artificial juice (powdered or bottled or boxed), and soft drinks.

Data processing and analysis were carried out using SPSS 13.0 software. To determine food frequency, a consumption index was calculated for each food item. Index “a” represents the number of times a given food is consumed per day. Thus, when the food was consumed daily, the number of times per day was considered (score = a). For foods consumed weekly, it was divided by seven (score = a/7); and for monthly consumption, it was divided by 30 (score = a/30); and, when not consumed, the corresponding value was zero. Then, to obtain the FVG and ULT group index, the frequency of consumption of each food was added and divided by the number of items in each group. When equal to or greater than one, it was considered that the participant consumed the respective food group daily. Finally, the consumption indices for each group (FVG and ULT) were transformed into quartiles, allowing a better assessment of central tendency and data dispersion. This variable was used for association analyses.

The consumption frequency index for each food item is detailed in Table 1, presenting mean values and standard deviations. Differences between analysis categories were assessed using the chi-square test (χ2), as outlined in the supplementary material (Supplement). Variables with a p value <0.2 were included in the multivariate analysis. Therefore, to verify the aspects independently associated with the consumption of FVG and ULT, multinomial logistic regression was performed. The lowest quartile served as the reference category, with p values <0.05 considered statistically significant (Tables 2 and 3). Quartiles Q3 and Q4 were considered as high and high-average consumption, respectively.

**Table 1:** Description of consumption of fruits/vegetables and ultra-processed foods by adults in Pernambuco, 2015/2016 (n=1,067).

FVG*	m (±sd)	ULT^†^	m (±sd)
Banana	0.44 (±0.47)	Margarine	0.77 (±0.66)
Orange	0.28 (±0.33)	Cream crackers	0.35 (±0.47)
Passion fruit	0.18 (±0.25)	Cornstarch and Maria biscuit	0.29 (±0.39)
Acerola	0.17 (±0.24)	Processed meats	0.24 (±0.31)
Apple	0.17 (±0.28)	Candies and sweets	0.17 (±0.38)
Watermelon	0.15 (±0.20)	Biscuit with filling or butter	0.16 (±0.31)
Papaya	0.12 (±0.24)	Artificial juice	0.30 (±0.42)
Raw salad	0.45 (±0.40)	Soda	0.25 (±0.39)
Carrot	0.24 (±0.32)	-	-
*Jerimum*	0.17 (±0.29)	-	-

FVG: fruits, vegetables, and greens; m: mean; sd: standard deviation; ULT: ultra-processed.

*added value of the 10 fruit/vegetable/greens items;

^†^added value of the 8 ultra-processed items analyzed.

**Source:** IV Pesquisa Estadual de Saúde e Nutrição (PESN-2015/2016).

**Table 2:** Associations between the consumption of ultra-processed foods and social, lifestyle, and metabolic factors of adults in Pernambuco, 2015/2016 (n=1,067).

Characteristics	OR* (95%CI^†^) Q2	OR (95%CI) Q3	OR (95%CI) Q4	p-value
Age				
20–29.99	1.282 (0.695–2.363)	1.946 (1.012–3.742)	3.797 (1.877–7.680)	0.005
30–39	1.701 (0.958–3.021)	1.867 (0.997–3.496)	2.650 (1.330–5.280)	
40–49	1.079 (0.598–1.948)	1.330 (0.704–2.510)	1.427 (0.691–2.946)	
50–59	Ref	Ref	Ref	
Housing area				
Rural	0.973 (0.613–1.546)	0.476 (0.288–0.786)	0.6 (0.356–1.011)	0.006
Urban	Ref	Ref	Ref	
Education				
≥complete high school	1.316 (0.798–2.170)	1.753 (1.052–2.922)	1.118 (0.657–1.903)	0.275
Complete elementary school/incomplete high school	1.506 (0.822–2.758)	1.860 (1.006–3.440)	1.469 (0.783–2.757)	
Never attended/incomplete elementary school	Ref	Ref	Ref	
Income (minimum wage)				
<0.5	1.888 (0.909–3.921)	3.211 (1.413–7.297)	3.469 (1.402–8.582)	0.044
0.5 to <1	1.616 (0.720–3.628)	2.115 (0.861–5.195)	3.226 (1.221–8.523)	
≥1	Ref	Ref	Ref	
Water treatment				
Filtered/boiled/strained	1.055 (0.615–1.809)	1.215 (0.703–2.098)	0.796 (0.441–1.434)	0.805
No treatment	1.215 (0.739–1.997)	1.124 (0.674–1.875)	0.958 (0.563–1.63)	
Mineral	Ref.	Ref.	Ref.	
Physical activity				
Very active	0.834 (0.355–1.963)	1.326 (0.522–3.374)	1.719 (0.646–4.578)	0.243
Active	1.454 (0.715–2.959)	1.767 (0.788–3.963)	1.998 (0.837–4.769)	
Irregularly active	1.703 (0.800–3.625)	1.947 (0.826–4.589)	1.504 (0.590–3.836)	
Sedentary	Ref	Ref	Ref	
Smoking				
No	1.268 (0.733–2.193)	0.823 (0.477–1.421)	1.203 (0.653–2.218)	0.436
Yes	Ref	Ref	Ref	
FVG quartiles*				
1	0.636 (0.366–1.106)	0.488 (0.277–0.861)	0.242 (0.131–0.447)	<0.001
2	1.295 (0.729–2.3)	1.143 (0.642–2.035)	0.836 (0.467–1.498)	
3	1.679 (0.957–2.947)	1.230 (0.692–2.188)	0.994 (0.557–1.774)	
4	Ref	Ref	Ref	
Hypertension^†^				
No	1.63 (1.018–2.61)	1.556 (0.948–2.556)	1.416 (0.833–2.406)	0.172
Yes	Ref	Ref	Ref	
Blood glucose^‡^				
<100	1.637 (0.971–2.759)	1.462 (0.845–2.529)	2.698 (1.378–5.283)	0.021
≥100	Ref	Ref	Ref	

FVG: fruits, vegetables, and greens; OR: odds ratio; 95%CI: 95% confidence interval.

*added value of the 10 fruit/vegetable/greens items;

^†^n=1,066;

^‡^n=866.

**Source:** IV Pesquisa Estadual de Saúde e Nutrição (PESN-2015/2016).

**Table 3:** Associations between consumption of fruits/vegetables/greens and social, lifestyle, and metabolic factors of adults in Pernambuco, 2015/2016 (n=1,067).

Characteristics	OR (95%CI) Q2	OR (95%CI) Q3	OR (95%CI) Q4	p-value
Age				
20–29.99	0.88 (0.45–1.71)	0.84 (0.43–1.64)	0.56 (0.29–1.11)	0.391
30–39	1.00 (0.53–1.92)	0.81 (0.42–1.56)	0.87 (0.46–1.64)	
40–49	1.51 (0.75–3.03)	1.36 (0.68–2.71)	1.28 (0.64–2.54)	
50–59	Ref	Ref	Ref	
Housing area				
Rural	0.83 (0.48–1.43)	0.79 (0.46–1.37)	0.81 (0.46–1.41)	0.835
Urban	Ref	Ref	Ref	
People in the household				
Up to 4	1.21 (0.79–1.84)	1.45 (0.94–2.24)	1.65 (1.07–2.55)	0.119
5 or more	Ref	Ref	Ref	
Education				
≥complete high school	1.39 (0.82–2.36)	1.63 (0.95–2.78)	1.71 (1.00–2.91)	0.428
Complete elementary school/incomplete high school	0.88 (0.48–1.60)	1.24 (0.69–2.23)	1.19 (0.66–2.17)	
Never attended/incomplete elementary school	Ref	Ref	Ref	
Income (minimum wage)				
<0.5	0.31 (0.09–1.04)	0.14 (0.04–0.44)	0.18 (0.06–0.57)	0.001
0.5 to <1	0.63 (0.17–2.29)	0.27 (0.08–0.90)	0.34 (0.09–1.15)	
≥1	Ref	Ref	Ref	
Waste				
General network	1.13 (0.65–1.96)	1.28 (0.71–2.31)	1.01 (0.58–1.75)	0.182
Septic Tank	1.2 (0.69–2.14)	2.12 (1.18–3.81)	1.19 (0.6–2.11)	
Others	Ref	Ref	Ref	
Water treatment				
Filtered/boiled/strained	1.09 (0.62–1.91)	1.01 (0.57–1.79)	0.89 (0.51–1.58)	0.450
No treatment	0.87 (0.52–1.44)	0.77 (0.46–1.28)	0.57 (0.34–0.95)	
Mineral	Ref	Ref	Ref	
Physical activity				
Very active	2.49 (0.92–6.74)	2.39 (0.95–6.06)	2.45 (0.89–6.73)	0.094
Active	1.26 (0.55–2.89)	0.82 (0.38–1.75)	1.42 (0.61–3.28)	
Irregularly active	1.38 (0.58–3.32)	0.86 (0.38–1.95)	1.13 (0.46–2.77)	
Sedentary	Ref	Ref	Ref	
Smoking				
No	1.04 (0.58–1.87)	1.42 (0.77–2.61)	1.09 (0.60–2.00)	0.667
Yes	Ref	Ref	Ref	
Alcohol consumption				
No	0.98 (0.63–1.53)	0.58 (0.38–0.91)	0.97 (0.62–1.53)	0.031
Yes	Ref	Ref	Ref	
ULT quartiles*				
1	0.30 (0.12–0.56)	0.24 (0.13–0.46)	0.24 (0.13–0.45)	<0.001
2	0.59 (0.32–1.08)	0.66 (0.36–1.21)	0.39 (0.21–0.71)	
3	0.71 (0.38–1.32)	0.66 (0.35–1.23)	0.51 (0.27–0.95)	
4	Ref	Ref	Ref	
BMI^†^				
<25	0.63 (0.37–1.07)	0.53 (0.31–0.92)	0.69 (0.40–1.21)	0.047
25–29.9	0.58 (0.34–0.99)	0.91 (0.54–1.52)	0.97 (0.57–1.64)	
≥30	Ref	Ref	Ref	
Hypertension^‡/^				
No	0.86 (0.50–1.47)	0.55 (0.33–0.94)	0.88 (0.51–1.52)	0.120
Yes	Ref	Ref	Ref	
Blood glucose^§^				
<100	2.10 (1.12–3.95)	1.32 (0.78–2.37)	1.05 (0.58–1.87)	0.078
≥100	Ref	Ref	Ref	

ULT: ultra-processed; BMI: body mass index; OR: odds ratio; 95%CI: 95% confidence interval.

*added value of the 8 ultra-processed items analyzed;

^†^n=1,014;

^‡^n=1,066;

^§^n=866.

**Source:** IV Pesquisa Estadual de Saúde e Nutrição (PESN-2015/2016).

To assess metabolic changes, self-reported hypertension, fasting blood glucose, and Body Mass Index (BMI) were evaluated. Knowledge of hypertension was determined using the question “has any health professional ever told you that you have high blood pressure?” Individuals were considered aware of the condition if they reported a previous diagnosis by a doctor or health professional.

For blood glucose analysis, blood samples were obtained following a 10-hour fast and measured using the Accutrend GCT equipment, which provides immediate readings following venipuncture. The values used to characterize the data were based on the Official Guideline of the Brazilian Diabetes Society^21^. According to this guideline, fasting glycemia levels were classified as: normal <100 mg/dl, pre-diabetes between 100 and 125 mg/dl, and diabetes >125 mg/dl.

To assess weight, adults were weighed using a digital scale (Model Tanita – BF-683 w/UM028 3601) and height was measured with a portable stadiometer (Altura Exata Ltda.), with an accuracy of 1 mm throughout its entirety. Both measurements were conducted in accordance with international recommendations^22^. Weight and height were then used to calculate BMI (kg/m^2^), considering overweight for values ranging from 25 to 29.99 kg/m^2^ and obesity ≥30 kg/m^2 21^.

This work was approved by the Ethics and Research Committee of the Academic Center of Vitória de Santo Antão, complying with the ethical precepts of Resolution no. 466, of December 12, 2012, of the National Health Council. Given its association with another project, authorization was obtained to utilize the requested data.

## RESULTS

The study included individuals aged 20 to 59 years old, with a total of 1,067 participants analyzed. The majority of the population was female (62.9%), and 72.8% resided in urban areas. Approximately 49.5% of participants never attended school or had incomplete primary education; and 75.5% lived with a *per capita* family income of less than half the minimum wage. Waste disposal was associated with the general network for 41.2% of individuals, while 39% had a covered septic tank. The majority (62.9%) lived with up to four people in the household. Regarding lifestyle factors, 55.8% were classified as active, 83.8% did not smoke, and 64.6% did not consume alcohol. Regarding metabolic aspects, 33.6% and 27.5% were overweight and obese, respectively; 20.9% self-reported a medical diagnosis of hypertension; and 11.9% had blood glucose levels greater than or equal to 100 mg/dl (Suppl.).

According to Table 1, the main foods consumed in the FVG group were banana (0.44; ±0.47) and raw salad (0.45; ±0.40), with a frequency of consumption around three times per week; in addition to orange (0.28; ±0.33), with an average intake of twice a week. In the ULT group, the most consumed items were margarine (0.77; ±0.66), consumed more than five times a week; cream cracker biscuit (0.35; ±0.47); and artificial juice (0.30; ±0.42), consumed on average two to three times a week. None of the foods evaluated had a consumption index equal to or greater than one, indicating that none were consumed daily or more than once a day.

The lowest quartile of consumption (Q1) of FVG and ULT grouped people with a frequency of consumption index lower than 0.117 and 0.178, respectively. On the other hand, in the highest consumption quartile, the index was greater than or equal to 0.330 for FVG and 0.419 for ULT. This finding indicates a lower consumption level than expected for FVG, as it remains far from daily consumption (equal to 1). However, it can be considered high for ULT, as consumption of this food group should be avoided.

In the bivariate analysis (Supl.), the variables age, living area, education, level of physical activity, quartiles of FVG, hypertension, and blood glucose were associated with the consumption of ULT. However, the multivariate model revealed independent associations with three sociodemographic variables, one lifestyle factor, and one metabolic factor, as described in Table 2. The consumption of ULT in the last quartile, when compared to the first, is three times higher in younger individuals (OR=3.79; CI=1.87–7.68) and among those with lower income (OR=3.47; CI=1.40–8.58). Additionally, it is more than double in people with controlled glycemia (OR=2.69; CI=1.38–5.28). Living in a rural area, compared to an urban area, reduces the chance of having a medium-high consumption of ultra-processed foods by 52% (OR=0.48; CI=0.28–0.79). Furthermore, lower consumption of FVG is related to lower consumption of ultra-processed foods (OR=0.30; CI=0.12–0.57).

The variables housing area, number of people in the household, education, income, water treatment, smoking, alcohol consumption, ULT, and BMI quartiles were associated with the consumption of FVG, in the bivariate analysis (Supl.). However, the multivariate model showed an independent association with two lifestyle variables, one sociodemographic factor, and one metabolic factor, as described in Table 3. Individuals with lower income have 86% and 82% less chance of having a high-average (Q3) (OR=0.14; CI=0.04–0.44) and high (Q4) (OR=0.18; CI=0.06–0.57) consumption of FVG, respectively. However, this consumption was higher among those who drank alcohol, consumed more ULT, and had BMI>25 (Table 3).

## DISCUSSION

Sociodemographic aspects such as age and area of residence, along with metabolic issues like blood glucose levels, were identified as determinants of ULT consumption. On the other hand, higher consumption of FVG was associated with higher BMI and alcohol consumption. Income emerged as a common determinant, although the direction of the association was different, that is, inversely associated with ULT intake and directly related to FVG intake. ULT intake surpassed FVG intake in both the lowest and highest quartiles of the consumption index.

Baker et al.^23^ observed a considerable expansion in the types and quantities of ULT sold worldwide. This ULT consumption tends to increase as countries become wealthier^24^, which partially explains the rise in calorie consumption from these foods. In the present study, ULT intake exceeded that of FVG in both the lowest and highest quartile of the consumption index. This finding underscores the acceleration in preference and access to ULT at the expense of fresh and minimally processed foods.

According to the findings of Canuto et al.^25^, in Brazil, individuals with higher income tend to consume more FVG and meat, alongside an increased consumption of ultra-processed foods within this same economic group. This dietary pattern, characterized as “Double,” denotes the coexistence of fresh foods with a high degree of processing, and is more prevalent in the highest socioeconomic strata^25^, reinforcing the relationship between income and a more diverse diet, despite not guaranteeing healthiness. The present study corroborates the relationship between income and FVG shown in the critical review mentioned earlier^25^. However, it reveals an inverse relationship with ULT, indicating that the burden of high consumption of highly industrialized products is transferred to more economically vulnerable groups.

In England, lower socioeconomic groups tend to have lower daily consumption of fruits and vegetables^24^. Similarly, in Brazil, inequality in the consumption of fruits and vegetables has increased over a period of 12 years^26^. Purchases of fruit decreased from 54.4 g per person and per day to 42.7 g, and vegetables went from 49.7 g to 37.4 g, over a span of 10 years. Interestingly, the Southern region of Brazil showed the largest acquisition of fruits and vegetables^27^. These findings confirm the significant challenge of achieving an adequate and healthy diet, reinforcing the continued inaccessibility of such a diet for many individuals.

Data from the Behavioral Risk Factor Surveillance System (BRFSS) of the United States of America (2015) revealed that only 12.2% of respondents met the recommended daily fruit consumption (1.5 to 2 servings) and 9.3% met the recommended daily vegetable intake (2 to 3 servings)^28^. Although there were variations according to the state, young adults had overall lower rates of recommended fruits (18–30 years=9.2%/ ≥51 years=12.4%) and vegetables (18–30 years=6.7 %/≥51 years=10.9%) consumption compared to adults aged 51 years old or older.

In a UK cohort, adults in the highest quartile of ULT consumption, in addition to being younger, were more likely to live in more vulnerable areas^8^. Adults and Aged people from a nutrition clinic in the interior of Rio Grande do Sul^29^ also showed an inverse and significant correlation between age and the consumption of calories from carbohydrates (p=0.001) and lipids (p=0.0048) from ultra-processed foods. These studies, along with the current research, reinforce the need for health promotion policies targeted at the young population, who are the primary audience for ULT food marketing and advertisements.

Borges et al.^30^, when analyzing 650 commercial establishments selling food in Jundiaí, São Paulo, found that, in central areas, of medium and high-income, the number of establishments prioritizing the sale of ultra-processed foods is 5.6 times greater than those selling fresh food. In peripheral, medium- and low-income areas, this ratio increases to up to 22 times higher. The availability of lipids and energy, as well as the consumption of sweet and artificially sweetened drinks, is higher in urban areas, while carbohydrates and proteins are more present in the diet of rural areas^25^. The influence of geographic and socioeconomic inequality on the distribution of food stores and the types of products sold is significant, which may partially explain the higher consumption of ULT foods by individuals residing in urban areas found in this study.

In a cohort study carried out by Levy et al.^10^ in the United Kingdom, participants with the highest levels of ULT consumption had a 44% increased risk of developing type 2 *diabetes mellitus*, and the incidence of this morbidity increased by 12% for each 10-percentage-point increase in ULT consumption. However, the present study observed greater consumption of ULT by individuals without glycemic alterations and greater consumption of FVG in individuals with higher BMI, which can be justified by previous guidelines.

Evidence suggests that good eating habits often accompany other behaviors associated with a healthier lifestyle. Individuals who are more health-conscious, focusing on weight maintenance and regular physical activity, tend to have a higher consumption of fruits and vegetables^14,31^. However, in the present study, FVG consumption was higher among those who consumed alcoholic beverages, which suggests a potential compensatory mechanism and may warrant further investigations into the intention, quantity, and time interval of consumption.

Strategies such as the Strategic Action Plan for Coping with Chronic Diseases and Non-Communicable Diseases in Brazil 2021–2030^32^; the new version of the Food Guide for the Brazilian Population^33^; and the goals established through the United Nations Decade of Action on Nutrition, from 2016 to 2025^34^, are essential for triggering intensified efforts to understand and address social, demographic inequalities, lifestyle, and metabolic factors associated with the consumption of fresh and ultra-processed foods.

The limitations of the study are based on the potential biases inherent to the cross-sectional design, as it does not allow causal relationships to be inferred, since exposure and outcome are measured simultaneously. The use of an FFQ restricted the analysis of daily calorie intake; however, it facilitated the categorization of individuals’ typical food consumption patterns. Self-reported high blood pressure data lead to under- or overestimation of prevalence, yet the collection method is scientifically validated^35^.
