# Supplementary material for: Consumption of in natura and ultra-processed foods in adults: an analysis of social, metabolic, and lifestyle determinants
Source: Rev Bras Epidemiol. 2024 Apr 29;27:e240018. doi: 10.1590/1980-549720240018 (PMC11057841; doi:10.1590/1980-549720240018)
Supplement: Supplementary file 1 [file 1980-5497-rbepid-27-e240018-Material-suplementar.pdf]

Resultados – Arquivo Suplementar

Tabela Suplementar: Descrição da amostra quanto aos aspectos sociais, de estilo de vida e metabólicos e quartis de consumo de ultraprocessados e frutas/legumes/verduras em adultos pernambucanos, 2015/16 (N=1067).

Variáveis		Quartis consumo de ULT					Quartis consumo de FLV				
	Total	Q1	Q2	Q3	Q4	P valor	Q1	Q2	Q3	Q4	P valor
	n(%)	n(%)	n(%)	n(%)	n(%)		n(%)	n(%)	n(%)	n(%)	
**Sociais**											
**Idade (anos)**						<0,001					0,580
20-29,99	362(33,9)	67(18,5)	77(21,3)	92(25,4)	126(34,8)		96(26,5)	97(26,8)	94(26,0)	75(20,7)	
30-39	343(32,1)	61(17,8)	104(30,3)	93(27,1)	85(24,8)		82(23,9)	79(23,0)	90(26,2)	92(26,8)	
40-49	196(18,4)	57(29,1)	55(28,1)	49(25,0)	35(17,9)		41(20,9)	47(24,0)	54(27,6)	54(27,6)	
50-59	166(15,6)	67(40,4)	46(27,7)	30(18,1)	23(13,9)		42(25,3)	37(22,3)	41(24,7)	46(27,7)	
**Sexo**						0,465					0,326
Masculino	396(37,1)	154(23,0)	175(26,1)	162(24,1)	180(26,8)		166(24,7)	159(23,7)	167(24,9)	179(26,7)	
Feminino	671(62,9)	98(24,7)	107(27,0)	102(25,8)	89(22,5)		95(24,0)	101(25,5)	112(28,3)	88(22,2)	
**Área**						<0,001					0,001
Rural	290(27,2)	90(31,0)	91(31,4)	51(17,6)	58(20,0)		94(32,4)	71(24,5)	70(24,1)	55(19,0)	
Urbana	777(72,8)	162(20,8)	191(24,6)	213(27,4)	211(27,2)		167(21,5)	189(24,3)	209(26,9)	212(27,3)	
**Pessoas no domicílio**						0,817					0,001
Até 4 pessoas	671(62,9)	159(23,7)	182(27,1)	160(23,8)	170(25,3)		141(21,0)	157(23,4)	188(28,0)	185(27,6)	
5 ou mais	396(37,1)	93(23,5)	100(25,3)	104(26,3)	99(25,0)		120(30,3)	103(26,0)	91(23,0)	82(20,7)	
**Escolaridade**						<0,001					<0,001
≥2º grau completo	354(33,2)	60(16,9)	97(27,4)	103(29,1)	94(26,6)		58(16,4)	83(23,4)	105(29,7)	108(30,5)	
1º grau comp./2º grau incompleto	185(17,3)	29(15,7)	43(23,2)	52(28,1)	61(33,0)		46(24,9)	45(24,3)	47(25,4)	47(25,4)	
Nunca frequentou /1º grau incompleto	528(49,5)	163(30,9)	142(26,9)	109(20,6)	114(21,6)		157(29,7)	132(25,0)	127(24,1)	112(21,2)	
**Renda**						0,105					<0,001
<0,5SM	806(75,5)	181(22,5)	213(26,4)	208(25,8)	204(25,3)		230(28,5)	196(24,3)	194(24,1)	186(23,1)	
0,5 à <1SM	190(17,8)	45(23,7)	50(26,3)	41(21,6)	54(28,4)		27(14,2)	53(27,9)	52(27,4)	58(30,5)	

≥ 1SM	71(6,7)	26(36,6)	19(26,8)	15(21,1)	11(15,5)		4(5,6)	11(15,5)	33(46,5)	23(32,4)	
**Dejetos**						0,464					0,119
Rede geral	440(41,2)	94(21,4)	116(26,4)	115(26,1)	115(26,1)		103(23,4)	114(25,9)	112(25,5)	111(25,2)	
Fossa tampada	416(39,0)	106(25,5)	113(27,2)	90(21,6)	107(25,7)		97(23,3)	94(22,6)	126(30,3)	99(23,8)	
Outros (fossa rudimentar, curso d'água, etc.)	211(19,8)	52(24,6)	53(25,1)	59(28,0)	47(22,3)		61(28,9)	52(24,6)	41(19,4)	57(27,0)	
**Tratamento de Água**						0,08					0,001
Filtrada,fervida, coada	231(21,6)	56(24,2)	62(26,8)	57(24,7)	56(24,2)		60(26,0)	61(26,4)	60(26,0)	50(21,6)	
Sem tratamento	344(32,2)	96(27,9)	98(28,5)	74(21,5)	76(22,1)		106(30,8)	84(24,4)	86(25,0)	68(19,8)	
Mineral	492(46,1)	100(20,3)	122(24,8)	133(27,0)	137(27,8)		95(19,3)	115(23,4)	133(27,0)	149(30,3)	
**Estilo de Vida**											
**Atividade física (n=1067)**						0,022					0,051
Muito ativo	134(12,6)	38(28,4)	25(18,7)	34(25,4)	37(27,6)		23(17,2)	37(27,6)	42(31,3)	32(23,9)	
Ativo	595(55,8)	125(21,0)	157(26,4)	145(24,4)	168(28,2)		143(24,0)	139(23,4)	148(24,9)	165(27,7)	
Irregularmente ativo	268(25,1)	65(24,3)	82(30,6)	68(25,4)	53(19,8)		70(26,1)	72(26,9)	68(25,4)	58(21,6)	
Sedentário	70(6,6)	24(34,3)	18(25,7)	17(24,3)	11(15,7)		25(35,7)	12(17,1)	21(30,0)	12(17,1)	
**Tabagismo**						0,166					0,011
Não/ex	894(83,8)	200(22,4)	242(27,1)	222(24,8)	230(25,7)		203(22,7)	216(24,2)	242(27,1)	233(26,1)	
Sim	173(16,2)	52(30,1)	40(23,1)	42(24,3)	39(22,5)		58(33,5)	44(25,4)	37(21,4)	34(19,7)	
**Consumo de bebida alcoólica**						0,894					0,022
Não	689(64,6)	167(24,2)	180(26,1)	167(24,2)	175(25,4)		174(25,3)	174(25,3)	159(23,1)	182(26,4)	
Sim	378(35,4)	85(22,5)	102(27,0)	97(25,7)	94(24,9)		87(23,0)	86(22,8)	120(31,7)	85(22,5)	
**Quartis FLV**[*]						<0,001	-	-	-	-	-
1	261	93(35,6)	69(26,4)	55(21,1)	44(16,9)		-	-	-	-	-
2	260	52(20,0)	66(25,4)	71(27,3)	71(27,3)		-	-	-	-	-
3	279	55(19,7)	86(30,8)	68(24,4)	70(25,1)		-	-	-	-	-
4	267	52(19,5)	61(22,8)	70(26,2)	84(31,5)		-	-	-	-	-
**Quartis ULT**[**]		-	-	-	-	-					<0,001

1	252	-	-	-	-	-	93(36,9)	52(20,6)	55(21,8)	52(20,6)	
2	282	-	-	-	-	-	69(24,5)	66(23,4)	86(30,5)	61(21,6)	
3	264	-	-	-	-	-	55(20,8)	71(26,9)	68(25,8)	70(26,5)	
4	269	-	-	-	-	-	44(16,4)	71(26,4)	70(26,0)	84(31,2)	
**Metabólicas**											
**IMC**[1]						0,957					0,002
<25	362(33,9)	86(23,8)	92(25,4)	94(26,0)	90(24,9)		109(30,1)	94(26,0)	79(21,8)	80(22,1)	
25-29,9	359(33,6)	88(24,5)	100(27,9)	81(22,6)	90(25,1)		77(21,4)	73(20,3)	103(28,7)	106(29,5)	
≥30	293(27,5)	69(23,5)	75(25,6)	73(24,9)	76(25,9)		59(20,1)	81(27,6)	82(28,0)	71(24,2)	
**Hipertensão**[2]						<0,001					0,089
Não	843(79,0)	173(20,5)	225(26,7)	218(25,9)	227(26,9)		209(24,8)	213(25,3)	206(24,4)	215(25,5)	
Sim	223(20,9)	79(35,4)	57(25,6)	46(20,6)	41(18,4)		51(22,9)	47(21,1)	73(32,7)	52(23,3)	
**Glicemia** [3]						<0,001					0,063
<100	739(69,3)	167(22,6)	205(27,7)	185(25,0)	182(24,6)		175(23,7)	194(26,3)	189(25,6)	181(24,5)	
≥100	127(11,9)	54(42,5)	31(24,4)	28(22,0)	14(11,0)		39(30,7)	20(15,7)	34(26,8)	34(26,8)	

[*]Valor agregado dos 10 itens de frutas, legumes e verduras; [**]Valor agregado dos 8 itens ultraprocessados analisados; [1]N= 1014; [2]N= 1066; [3]N= 866.

Fonte: Pesquisa Estadual de Saúde e Nutrição (PESN), 2015-2016.
